# Supplementary material for: West Nile Disease Symptoms and Comorbidities: A Systematic Review and Analysis of Cases
Source: Trop Med Infect Dis. 2022 Sep 8;7(9):236. doi: 10.3390/tropicalmed7090236 (PMC9506265; doi:10.3390/tropicalmed7090236)
Supplement: Supplementary file 1 [file tropicalmed-07-00236-s001.zip › tropicalmed-1819977-supplementary/ALL SUPPLEMENTARY.pdf]

SUPPLEMENTARY FIGURES

Search Strategies

Pubmed (1,037 entries):

#1 (West Nile Virus [MH]) OR (West Nile [ti])

#2 (case reports [publication type]) OR (case reports[mh]) OR (case[tiab]) OR (diagnos*[tiab])

#3 (animals[mh]) NOT (humans[mh])

#4 (#1 AND #2) NOT #3

EMBASE (via Ovid, 1583 entries)

#1 exp west nile virus/

#2 west nile.ti

#3 #1 or #2

#4 exp case report/

#5 case.ti,ab

#6 diagnos$.ti,ab

#7 #4 or #5 or #6

#8 exp animals/

#9 exp humans/

#10 #8 not #9

#11 (#3 and #7) not #10

Figure S1. The search strategy terms as used in the Rayan platform for systemic studies for PubMed and EMBASE databases.

SUPPLEMENTARY TABLES

Table S1. Raw cases counts and statistical significance of symptoms between deceased and recovered patients' groups. Significance values in bold designates p-values <0.05.

	**Deceased patients (*n*=)**		**Recovered patients (*n*=)**		
**Symptoms**	**with symptom**	**without symptom**	**with symptom**	**without symptom**	**Significance level (*p*-value)**
altered mental status / confusion / delirium	51.00	46.00	136.00	206.00	**= 0.00805**
dizziness	3.00	94.00	20.00	322.00	= 0.28348
memory loss / disorientation	3.00	94.00	21.00	321.00	= 0.24486
cognitive diff / impaired consciousness / agnosia	6.00	91.00	33.00	309.00	= 0.29101
headache	22.00	75.00	132.00	210.00	**= 0.00367**
photophobia	1.00	96.00	18.00	324.00	= 0.07088
speech problems / dysarthria / aphasia	15.00	82.00	44.00	298.00	= 0.50891
hyporeflexia / areflexia / unresponsiveness	18.00	79.00	46.00	296.00	= 0.20932
malaise / numbness / dysesthesia	9.00	88.00	21.00	321.00	= 0.28071
walking-gait problems / poor balance	4.00	93.00	40.00	302.00	**= 0.02840**
seizures / status epilepticus	4.00	93.00	11.00	331.00	= 0.66502
fever/pyrexia (West Nile fever)	85.00	12.00	259.00	83.00	**= 0.01195**
cough / sore throat	5.00	92.00	12.00	330.00	= 0.45950
tremors / chills / rigors / sweats	27.00	70.00	84.00	258.00	= 0.51374
extremity weakness / paralysis / lesion	36.00	61.00	121.00	221.00	= 0.75392
fatigue / generalized weakness	26.00	71.00	102.00	240.00	= 0.98972
muscle weakness - stiffness	9.00	88.00	45.00	297.00	= 0.30561
myoclonus	4.00	93.00	20.00	322.00	= 0.51079
ataxia / dysmatria	1.00	96.00	12.00	330.00	= 0.20473
neck pain / stiffness	9.00	88.00	51.00	291.00	= 0.15464
abdominal pain	5.00	92.00	22.00	320.00	= 0.64466
myalgia	12.00	85.00	43.00	299.00	= 0.95782
arthralgia	5.00	92.00	10.00	332.00	= 0.28684
rash	5.00	92.00	50.00	292.00	**= 0.01287**
nausea / vomiting / emesis	33.00	64.00	97.00	245.00	= 0.28239
anorexia / weight loss	11.00	86.00	13.00	329.00	**= 0.00387**
diarrhea	16.00	81.00	45.00	297.00	= 0.40283
respiratory problem	10.00	87.00	35.00	307.00	= 0.98282
kidney/urine problem	7.00	90.00	9.00	333.00	**= 0.03348**
agitation / palpitation / tachycardia	3.00	94.00	8.00	334.00	= 0.67596
somnolence / lethargy / sleepiness / drowsiness	15.00	82.00	51.00	291.00	= 0.89357
coma	1.00	96.00	5.00	337.00	= 0.74758

Table S2. Raw cases counts and statistical significance of measurements between deceased and recovered patients' groups. Significance values in bold designates p-values <0.05.

CSF & Blood tests	Deceased patients (*n*=)		Recovered patients (*n*=)		Significance level (p-value)
	with abnormal readings	with normal readings	with abnormal readings	with normal readings	
protein (normal 20-45 mg/dL)	53	6	173	8	= 0.13622
glucose (normal 45–80 mg/dL)	14	16	20	79	**= 0.00030**
White blood cells/µL (normal 0-5)	55	7	183	14	= 0.14899
Red blood cells/µL (normal 0-5)	13	3	38	19	= 0.91892
White blood cells/µL (4000-10800)	4	7	11	16	= 0.39490

Table S3. Raw cases counts and statistical significance of comorbidities between deceased and recovered patients' groups. Significance values in bold designates p-values <0.05.

Comorbidities	Deceased patients (*n*=)		Recovered patients (*n*=)		Significance level (*p*-value)
	with comorbidity	without comorbidity	with comorbidity	without comorbidity	
WNV past infection	0.00	97.00	6.00	336.00	= 0.18984
hepatitis C	1.00	96.00	7.00	335.00	= 0.51022
herpes simplex virus	0.00	97.00	1.00	341.00	= 0.59491
bacterial infection	2.00	95.00	8.00	334.00	= 0.87200
(auto)immune disorder	6.00	91.00	14.00	328.00	= 0.38430
cancer	27.00	70.00	41.00	301.00	**< 0.0001**
transplant / transfusion	33.00	64.00	43.00	299.00	**< 0.0001**
diabetes	19.00	78.00	44.00	298.00	= 0.09598
cardiovascular problems	15.00	82.00	41.00	301.00	= 0.36626
hyperlipidemia	4.00	93.00	9.00	333.00	= 0.44531
hypertension	19.00	78.00	51.00	291.00	= 0.26796
stroke	1.00	96.00	2.00	340.00	= 0.63874
pulmonary infection / disease	5.00	92.00	5.00	337.00	**= 0.03147**
kidney / renal problem	7.00	90.00	20.00	322.00	= 0.62142
spinal problems	1.00	96.00	4.00	338.00	= 0.90981
neurological problem	0.00	97.00	334.00	342.00	= 0.12904
smoking	1.00	96.00	4.00	338.00	= 0.90981

Table S4. Raw cases counts and statistical significance of administered therapy agents between deceased and recovered patients' groups. Significance values in bold designates p-values <0.05.

**Administered therapy**	**Deceased patients (*n*=)**	**Recovered patients (*n*=)**	**Significance level (p-value)**
antibiotics	16	32	= 0.58845
intravenous immunoglobulin	8	23	= 0.48353
corticosteroids	10	15	= 0.24911
anticoagulants	0	3	= 0.24624
antiherpetic drugs	11	16	= 0.18962
immunosuppresants	8	8	= 0.06912
mechanical ventilation - intubation	5	3	**= 0.04252**
antiseizure drugs	1	3	= 0.80351
interferon	0	2	= 0.34678
plasmapheresis	3	1	= 0.05059

Table S5. Gender counts in deceased and recovered patients' groups. Sex ratio and mean age is reported

	**Sex**		**Sex Ratio**	
	**Female**	**Male**	**(Female/Male)**	**Age**
**Deceased**	37	63	0.59	54.54
**Recovered**	121	216	0.56	60.59

Table S6. Geographical origin of cases in deceased and recovered patients' groups.

	**North America**	**South America**	**Europe**	**Africa**	**Asia**	**Australia**	**N/A**
**Deceased**	61	0	6	0	4	0	30
**Recovered**	205	1	42	3	15	4	66
